# Steps on a journey to TB control in Solomon Islands: a cross-sectional, mixed methods pre-post evaluation of a local language DVD

**DOI:** 10.1186/s12914-015-0041-3

**Published:** 2015-02-03

**Authors:** Peter D Massey, Rowena Asugeni, John Wakageni, Esau Kekeubata, John Maena’aadi, John Laete’esafi, Jackson Waneagea, Vunivesi Asugeni, David MacLaren, Richard Speare

**Affiliations:** Hunter New England Population Health, 470 Peel St, Locked bag 9783, Tamworth, NEMSC 2348 Australia; University of New England, Armidale, Australia; Atoifi Adventist Hospital, Malaita, Solomon Islands; James Cook University, Brisbane, Queensland Australia; East Kwaio, Malaita, Solomon Islands; Tropical Health Solutions, Townsville, Australia

**Keywords:** Tuberculosis, Solomon Islands, Heath education, Culture, Language

## Abstract

**Background:**

In Solomon Islands many people with Tuberculosis (TB) have challenges in accessing services because of socio-cultural, geographic and health service reasons, resulting in delays in TB treatment and low detection rates. The purpose of this project was to (i) develop a local language audio-visual resource (DVD) about TB (ii) share this resource with people in remote villages and (iii) evaluate the process and outcomes.

**Methods:**

The project involved the development and evaluation of a DVD in local Kwaio language. The DVD included five short videos based on the Australian Respiratory Council TB Education Flipchart. The DVD also included short videos of: traditional music/chanting (ai’imae); drama that presented an allegory of TB; and a short documentary on the redevelopment of the local TB Ward.

A mixed-methods approach evaluated changes in TB knowledge and investigated the impact of the DVD.

**Results:**

The DVD was recorded and produced in March–June 2013 and screened in 41 villages and hamlets. The pre-post DVD survey was completed by 64% (255/400) of people who viewed the DVD in the villages. Pre-DVD survey responses showed a moderate to high knowledge about TB signs, symptoms and treatment but 76/255 (30%) stated TB was caused by sorcery and 85/255 (33%) incorrectly stated that TB medication should be stopped when a patient feels better.

The post-DVD survey showed a significant increase in people in coastal villages reporting (i) a 3-week cough would trigger a medical assessment and (ii) TB is mainly spread through the air. Statements that TB is not caused by sorcery increased post-DVD in both coastal and mountain villages, however belief in sorcery in mountain villages remained high at 20/70 (29%).

**Conclusions:**

The local DVD resource was developed within local cultural understandings and oral traditions of Kwaio people. Using modern but accessible DVD technology generated a lot of interest about the disease and the stories.

The project evaluation indicates that current delays in seeking treatment may be more due to socio-cultural and health service factors than awareness of the disease. Therefore the development of TB services, including TB education, which are culturally sensitive, remains important.

## Background

Tuberculosis (TB) remains a prevalent public health issue in Solomon Islands with a notification rate of 66/100,000 population in 2012 [1]. TB is particularly important in the remote area of East Kwaio on the island of Malaita, where TB rates of 115/100,000 population have been reported and with a case detection rate of just 50% [2,3]. Access to health services for TB treatment in this area is challenging for many people because of many socio-cultural, geographic and/or health service reasons. This results in delays in seeking TB treatment and low case detection rates [4].

The delay in seeking treatment is one of the main drivers of the continued spread of TB in the community in many settings and including in Solomon Islands [5]. In neighbouring Papua New Guinea cultural beliefs combined with limited knowledge and awareness about early symptoms of TB contribute to why people present late [6]. Higher TB case detection rates have been reported in other developing settings when the understandings of cultural beliefs about TB are included in health programs [7-10].

In the Pacific Islands oral traditions are important in communicating knowledge, ideas and meanings and are valuable adjuncts to more formal western scientific sources of information [11]. There are more than 1000 local languages across the Pacific and people are increasingly using their own local languages in new aspects of their lives in addition to official, often colonial, languages such as English or French. Local languages are now being used to combat diseases and build environmental sustainability, when in the past this was mainly in the official language [12]. It has been reported that people from local language groups, who use local languages in their everyday life, can be more vulnerable to diseases such as TB, HIV/AIDS and malaria. This may be in part because the English, French or other official language used in health resources does not carry the depth of meaning needed to bring about change for people whose lives are based in another language [12].

The importance of visual and audio materials in local language for people with low literacy levels has also been highlighted in health education work in South Africa [13]. In that setting health understanding of TB was improved by working with rural health workers, local languages and mobile technology [13]. However, health education information that is translated from one language to another can be complicated because not all health concepts can be translated from one language to another or one culture to another. Recent work in Bangladesh found that some health resources had no cultural relevance to local people when translated from English into Bangla, even though they had been translated by competent translators [14].

Health education resources in the local Kwaio language are virtually non-existent despite many health challenges present in the area. In 2013, the TB team at Atoifi Adventist Hospital (AAH), in collaboration with public health researchers from Australia received a small grant (AUD$18,800) from the Australian Respiratory Council (ARC) to develop local language audio-visual resources about TB with communities in this isolated region of Solomon Islands. The funding enabled a Project Coordinator to work full-time with the support of a Kwaio Cultural Advisor (who is also a Village Health Worker), Kwaio Translation Advisors, Kwaio Chiefs and supported by public health researchers from Australia. This project is part of longer-term research capacity strengthening program in the area [15]. The capacity building aspects of the evaluation of the project were supported in part by the WHO Special Programme for Research and Training in Tropical Diseases (TDR).

Much of the recent research into delays in diagnosis in low resource settings, such as Solomon Islands, indicate numerous factors are involved: low educational level; low income; gender; rural life; unemployment; ageing; misunderstanding about the microbial cause of tuberculosis; low awareness of TB; incomprehensive beliefs; self-treatment; and stigma as associated community or individual factors. Health system factors include: low levels of health care coverage and consultation with a traditional healer or a non-skilled professional [5,16,17]. Very little has been reported in the Pacific context about TB at all, and even less on strategies to reduce delays in diagnosis.

The project aimed to: increase the TB case detection rate in East Kwaio through the development of a local language audio-visual resource for TB awareness; share this with people in remote villages and hamlets; evaluate the impact of the resource with local communities; and to describe key issues for further consideration. This paper aims to describe the development of the local language audio-visual resource and its evaluation. Changes to the TB case detection rate will be investigated at a later date.

## Methods

A reference group and governance structure were formed to direct the project in March 2013. The group of 12–15 people included: Chiefs from the coastal and mountain areas; local church leaders of various denominations; National TB Program staff; Australian public health researchers; and, leaders from the Atoifi Adventist Hospital and Atoifi Adventist College of Nursing. The reference group met regularly to review progress and advice on aspects of the DVD development and evaluation. The project was overseen day-to-day by the Director of Nursing. An important principle of the project was to hold to decolonising methodologies [18] and apply a participatory action framework [19] to ensure that Kwaio voices and Kwaio communities were central to the development and implementation of the project [4].

The content of the ARC “*What is TB*” flip chart was initially translated from English into the local Kwaio language, by the TB Coordinator. This translation occurred with the close involvement of Chiefs and the Cultural Advisor to ensure the integrity of the material. The flip chart contains information about what TB is, signs and symptoms of TB, how it is spread and how it is treated. Scripts and scenes for five small videos were then developed based on these translations. In addition ideas for three ‘bonus’ tracks were developed that included:

- traditional music & chanting (ai’imae) to accompany video of a person acting as a TB patient presenting to hospital;
- a drama that presented an allegory of TB being a rogue enemy that people needed to work together to defeat;
- a documentary style update on the redevelopment of the TB Ward at AAH to be more culturally appropriate for Kwaio people [2].

The videos were filmed using a small digital camera (Panasonic Lumix Tough DMCFT4GNA) funded by the grant and featured local health workers and community people. Editing, using Adobe Photoshop and Premier Elements 11, and production of DVDs were completed at AAH on an ASUS Notebook JBX. The DVD was then taken to more than forty villages and hamlets in the coastal and mountain regions of East Kwaio. Portable DVD players (Phillips PD9030), a solar panel and battery-powered speakers (purchased subsequently) enabled the people living in very remote villages & hamlets to see and hear the videos. There are no roads or electricity in any of the villages or hamlets. To show the DVD the TB team travelled by canoe to the coastal villages and walked along treacherous mountain trails, sometimes for several days, to mountain villages/hamlets.

A mixed-methods approach was taken to evaluate changes in knowledge about TB and more broadly how the DVDs were accepted. A written or oral survey was conducted before and after the DVD was displayed at each village. The survey questions asked basic yes/no questions about TB based on the content of the World Health Organization information sheet on TB [20]. The survey also collected basic demographic information. Each survey was entered into a Microsoft Excel spreadsheet and correct responses were noted. Proportions of respondents by location type (coastal/mountain) were calculated. Associations, using the Chi-square test, were investigated between the responses and the demographic variables of education and location. The proportion of respondent’s answers changing from incorrect pre-DVD to correct post-DVD, for each question, was tested using the McNemar significance test for probability, with significance set at <0.05.

Chiefs from the mountain areas, the Cultural Advisor, the TB Coordinator of the AAH participated in the structured interviews, and along with the Australian public health researchers, the analysis and interpretation of the results. The interviews were digitally recorded and analysed by the group using a deductive approach to answer specific questions from the data. The main questions were about the role sorcery plays in communities especially in relation to TB. Also factors about TB treatment and the DVD were extracted from the data. The interpretation was done in Kwaio language or Solomon Islands Pijin and translated into English when there was agreement across the group on the main issues and which quotes to use. In December 2013 results from the pre and post survey were presented back to village people at an open meeting adjacent to the weekly market at Atoifi.

External ethics approval was not required for this type of evaluation of a health education program. The AAH Administrative Committee and the Reference group supported the project. Co-authors on this paper include Chiefs who participated in the design of the TB DVD project, translation of material and interpretation of results. The Chiefs gave oral approval to participate. Participants in the survey gave oral consent and responded to the surveys voluntarily. Written consent was not used as literacy skills are low in this setting.

## Results

The results describe the development of the local language audio-visual resource and the evaluation. The TB DVDs were developed as planned during 2013, with five short videos about TB from the ARC TB flip-chart plus the three ‘bonus’ tracks. The DVDs were screened and evaluated at TB awareness events in forty-one villages or hamlets to approximately 400 people. More than 800 people in total watched the DVDs at the awareness events, amounting to just under 10% of Kwaio speakers in the region. Sixty-four percent (255/400) of the people within the villages who saw the DVD, volunteered to complete the pre-post DVD evaluation survey. The method aimed to sample 10 people from each village/hamlet. Many villages and hamlets did not have 10 adults but a total of 255 surveys were completed. Of the survey group 183 were from coastal villages and 70 were from mountain hamlets or villages, two people had no information recorded on location. Men comprised 51% and women 49% of the people who took part in the evaluation (Table 1).

**Table 1:** **Number, location, gender and age of people who took part in the evaluation of the DVD, East Kwaio, 2013**

	**Age group**	**Number**	**Location**	**Age group**	**Number**	**Totals**
Coastal Males	0-19	27	Mountain Males	0-19	8	35
	20-49	59		20-49	13	72
	50+	10		50+	10	20
	Unknown	2		Unknown	1	3
Coastal Females	0-19	28	Mountain Females	0-19	5	33
	20-49	51		20-49	24	75
	50+	5		50+	9	14
Unknown males	Unknown age	1	Unknown females	Unknown age	2	3
Totals		183			72	255

There were a variety of community roles reported by participants in the evaluation, including: Chiefs; church leaders; teachers; and village/hamlet members. The roles were similar for participants from both the coastal and mountain areas. The formal education level of the people who took part in the evaluation differed in the coastal and mountain areas. A significantly higher proportion (54/70) of the people in the mountain areas had no formal education compared to (14/183) people on the coast (p = 0.001) Table 2.

**Table 2:** **Education level by location of people who took part in the evaluation of the DVD, East Kwaio, 2013**

**Location/education**	**None**	**Primary**	**Secondary**	**Tertiary**	**Unknown**
Coastal	14 (8%)	79 (43%)	74 (40%)	1 (1%)	15 (8%)
Mountain	36 (51%)	11 (16%)	5 (7%)	0	18 (26%)
Unknown location		1			1

No formal education and living in a mountain hamlet were significantly associated (p = 0.0001) with reporting sorcery as the cause of TB pre and post the DVD.

After watching the DVD there was a significant (p = 0.003) increase in the proportion of coastal people reporting that a 3-week cough would trigger an assessment at the clinic or hospital (Table 3). Significant changes in correct responses to the knowledge survey were also seen post-DVD for knowing that TB is spread mainly through the air; not mainly spread on hands, food and clothes; and that TB is not caused by sorcery (Table 3).

**Table 3:** **TB knowledge correct responses pre-post DVD, by location, with p-values, East Kwaio, 2013**

**TB knowledge area**	**Area**	**Pre-DVD survey correct/Post-DVD survey correct**					
		**Yes/Yes**	**Yes/No**	**No/Yes**	**No/No**	**Exact McNemar significance probability**	
						p < 0.05 *	
TB caused by germ	Coastal	177	1	5	0	0.22	
	Mountain	58	2	7	3	0.18	
TB not caused by sorcery	Coastal	133	7	33	8	0.00	*
	Mountain	32	3	18	17	0.00	*
Person-person spread	Coastal	173	3	4	2	1.00	
	Mountain	65	2	1	2	1.00	
TB is spread through the air	Coastal	173	0	7	0	0.02	*
	Mountain	62	0	6	0	0.03	*
TB not spread easily on hands	Coastal	146	5	24	2	0.00	*
	Mountain	54	2	12	1	0.01	*
TB not spread easily on food	Coastal	29	5	87	57	0.00	*
	Mountain	23	3	21	22	0.00	*
TB not easily spread on clothes	Coastal	129	4	35	9	0.00	*
	Mountain	44	5	11	9	0.21	
Check cough after 3 weeks	Coastal	140	3	37	1	0.00	*
	Mountain	48	7	7	8	1.00	
TB can be cured	Coastal	175	3	5	0	0.73	
	Mountain	64	1	4	0	0.38	
Don't stop TB treatment early	Coastal	100	15	45	19	0.00	*
	Mountain	44	4	16	5	0.01	*
Not ok to smoke with TB	Coastal	170	6	4	2	0.75	
	Mountain	59	6	3	0	0.51	

*Significant difference pre-DVD to post-DVD.

After watching the DVD 43/255 or 17% (95% CI: 12.7%-22%) still reported (incorrectly) that it is ok for people with TB to stop taking the TB medicine when they felt better. No significant difference was found between coastal and mountain people for this factor. No further associations were found between TB knowledge and gender, community roles, education levels or location.

From the interviews with the Chiefs from the mountain areas and feedback at the community meeting the following three main issues were identified as being reoccurring themes: 1. Use of local language and the DVD; 2. Belief in sorcery; and 3. Discontinuing TB medication.

1. **Use of local language and the DVD:** Having the TB DVDs recorded in the local language was a key feature of the project. An important mountain Chief and co-author explained this by stating:

*“Inau nga le`anga ngai ai inau ku madafia ai na fatalana nga arikwao, ngai lo`oo gwa`a ka agea ma gwa`a inau me le`a agu. Gwaa `ua taa `ola ka iria ma nau ku “Fatanga lo`oo ku su`ai.” Ngai lo`oo nau ku laenia `akui su`ai. Gwa`a `ola imooru moru agea mai, ma moru agea fana tee `Ingelesi, ma gwa`a inau ma taku boobolosia mola. Ma alata imoru moru agea maka leka mola na languisi, gwaa `ola `uutaa ma inau taku bi`i fata i suria te`e bi`i le`a agu te`e wataga agu. Ngai lo`oo nau ku laenia ai. Na fui`ola agu lee `ilo`oo, suria `ola gurui arua fana languisi [inaudible: `ola agu lo’oo?…”*

Translation:

Me, what I think is good about what he said, if he [puts it into Kwaio] I like that. Anything he says, I’ll say, “This is a language I understand”. I like that because I want to understand it. Anything you guys do here, if you do it in just English, even I won’t grasp it. But if you put it all in language, then anything you talk about I’ll be able to talk about, and only then will it be satisfactory for me, be clear to me. That’s why I like it.

*Naku agasia te’e fate’enilai lo’ori miri agasia, me’e miri agasia ato amiri sua’ai. (JL)*

*I think seeing it (the DVD) once; we couldn’t fully understand it.*

*Nakumadafia amoru amuru agea ani taua nga mobile lo’o miri to’o ai meru amiri aga agasia, amiri fa’a nanaua na rurua sika’a ameru ma koko’o ameru, gila ame sua na ninginga I Atoifi malongo lana ta’a muru fafataisuria. Naku madafia amuru arua ubulana mobile lo’ori miri riringim ta’a ame’eru te’efou gilangai Honiara. Na manatalagu eino’ona ngai ani laoa fame’ru ami agasia meru te’efou anilea ana manatalamuru ani fola’a suria (JW)*

*I recommend that this video can be put into our mobile phone that this can be accessible. So we can be able to show and teach our children as well as our older people who were unable to make it to Atoifi or having the privilege to that information. I suggest that this video should be downloaded into our mobile phone that we used to call our wantoks in Honiara. I hope this is a good way of doing it that we would be happy about.*

2. **Belief in Sorcery:** The Chiefs discussed sorcery and there was strong agreement that it is common belief amongst the people that TB is caused by sorcery. This belief would likely delay people seeking treatment for a cough illness or other TB symptoms.

*What is in the mind of people when they saw or observed somebody with cough and losing weight that straight away they think and believe that it was sorcery or that particular devil [ancestral spirit]. (Directly in English)*

*Taem pikinini or man hemi siki lo bus nomata TB or wat kain siki definitli olketa no stap lo hospital ia, totali no. Hospital hemi the last ting. Okleta bae foloim proses, blo olketa.....givim go proses ia hemi gud, olketa stap nao. But gogo nonuf hem noa bae olketa cum lo hospital nao....dat wan hemi deli laef blo olketa, hem noa woka blo olketa olowei. Fo iumi go in lo olketa na lelebet link wetem olketa hem nao wat iumi bae doim.*

*Translation:*

*When a child or adult is sick in the mountains, if it is TB or whatever sickness, definitely they will not come to the hospital – totally no. The hospital is the last thing. People will follow their process, it belongs to them.....they will follow the process, if it works, then they will stop there. But, if they follow the system and it does not work, that is when people will come to the hospital. This is the daily life of people, this is what people do all the time. For us to go and make a link with people, then that is what we should do.*

3. Discontinuing TB medication: Discontinuing the use of medication when people felt better was described as the normal way that people managed traditional and modern treatments. This is a serious problem for TB medications and the risk of TB resistant strains developing.

*In our society is when we get these drugs, like for 6 months drug, and we take one week drug for starting and then feel better and we still we got the drug, then we don’t want to drink it because we keep the drug again for when we are sick again………..we want to keep the drug for us to protect us. So when we are sick again we [have] got the drugs at the same time, so we don’t try to spoil (waste) the drug. Usually this is what we are doing. (EK Directly in English)*

*We must work with the TB team and make some kind of system to make the people drink the drug to complete the dose. This is the input that we must work on to tell the people about trying to complete the dose. (EK Directly in English)*

## Discussion

Tuberculosis remains a key health issue of the people in the East Kwaio area of Solomon Islands. Improving the surveillance and response to this infectious and serious illness will enable important health gains to be made. This project has shown some of the value of developing health promotion resources that are embedded in the local language, culture and understandings. The development of the DVD provided a focus for increasing TB awareness in the health service and the people of the area. The evaluation has shown that the use of the DVD has somewhat increased levels of understanding about TB, but some issues need ongoing discussion and understanding between health professionals and communities.

The large majority of people had a moderate to high level of knowledge about some aspects of TB, in particular in relation to TB being a germ that is mostly spread through the air from infectious people. However, many people stated that TB was also caused by sorcery and a third also believed that TB medication should be stopped when a patient feels better rather than complete the full course of treatment. People with a higher level of formal education generally had a higher level of TB knowledge. This result is similar to other studies in settings with limited educational opportunities and diverse cultural beliefs [8,21,22]. A low level of knowledge that bacteria, and not sorcery, is the cause of TB described in this study, has also been reported as an important factor in treatment seeking delays and attitudes from other developing settings [23-25]. In a West African study patients generally described TB as a natural and/or magical disease. They highlighted the need for culturally sensitive TB health education in which traditional/religious perceptions and practices are not neglected, but identified as part of the cultural context that TB education messages would be interpreted within [26].

Local perceptions and beliefs about TB transmission, treatment and care can delay presentation for TB at medical facilities such as AAH. It has been noted previously that people may utilize more culturally embedded explanations of the disease and traditional healers who are more easily accessible, instead of hospital-based health care [4]. This has also been described in neighboring Vanuatu where seeking health care from traditional healers is the norm in communities that have a strong tradition of traditional healers and spiritual causation of illness [27]. The development of TB services, including TB education, that are culturally sensitive towards local people is recognized to be vitally important. Significant steps have been made towards this approach already in East Kwaio that include the building of a new TB ward that is located away from the maternity ward and providing food for inpatients with TB [2]. The maternity ward is a culturally taboo place for many people in East Kwaio. Having the TB ward located away from the maternity ward is therefore essential for equity of access for all Kwaio people.

The strengths of this ongoing project exist across the project. This is evident through the deep level of engagement with community leaders and communities that enabled: the development of the DVD; the filming of the DVD; the showing of the DVD; and the evaluation methods all to be centred on oral communication. Story-telling and discussion are integral parts of Pacific cultures [11], and this project connected with this central component to life in remote villages. One leader displayed the deep level of local ownership of the project when he said that he was “going to take *our* work up and tell everyone about it” (JW).

In addition to the strengths of oral communication the project and evaluation were conducted in local language and this was the first time that a DVD had been recorded in the local language. DVD technology is only very new into remote areas of Solomon Islands including in the project setting where there is no television, nor radio, nor electricity. The novelty factor was high and drew much interest to the DVDs and to TB as a result.

The mixed methods approach of the evaluation enabled not only measure of the change in knowledge but also an understanding of key aspects of how people understand and engage with TB as a social, cultural and disease phenomenon. Together these parts of the evaluation help to inform the next steps on the journey to control TB in this setting.

The evaluation of the DVD project has shown that enabling local health workers and communities to independently create health stories from within their own local communities facilitates a greater connection with people. Not only the health content but the people featured on the DVD were local people and local health workers. Communities being able to hear their own language and see their own people heightened the connection with the DVD. This connection is grounded also in the inclusion of local cultured understandings within the development and content. Similar work in South Africa has demonstrated this to be an effective approach to address TB [13]. The project in South Africa involved the development and use of mobile phone videos about HIV and about TB. The use of this technology brought credibility and trust to the message, whilst increasing interest and pride in the health care workers. This was also similar to the experience in the current project in East Kwaio. The story described in the South African project of a person with TB symptoms seeking health care, as a result of seeing how germs spread on a video, also occurred in East Kwaio. To facilitate the ongoing impact of the DVDs on health seeking behavior, the content will need to continue to be shared and discussed. Using mobile phone videos may be a way to achieve this.

As a result of the high level of community connectedness, and following on from public viewing and the evaluation of the DVD, local Chiefs are now discussing local spiritual beliefs about TB and its transmission. Some Chiefs now plan to work closely with the people who intercede with the specific ancestral spirits that are believed to cause TB to reduce this occurring. Likewise people who believe in healing by spiritual intervention following Christian prayer and other introduced beliefs are being encouraged to talk about the germ that causes TB and the importance of medical diagnosis and treatment. The World Health Organisation has acknowledged through a number of resolutions, the important role of traditional healers in health care [28]. Traditional healers have also been described as potential TB treatment supervisors for many years, but with issues noted about beliefs and understanding of the disease process [29]. A very pragmatic approach is also being taken with the TB team at AAH. This pragmatic approach asserts that even if people believe in spiritual causes or treatments of TB, as long as they attend a clinic for assessment as well, and take the TB treatment as prescribed, then the outcomes may be improved.

The presentation of the DVD in local language has proven to be so popular that people have requested that the DVD be presented at every hamlet in the Kwaio mountains. The DVD has also been requested to be seen in the West Kwaio area which is outside of the AAH catchment. The West Kwaio people and other nearby language groups also have high levels of TB. Having the DVD available on mobile phones would assist the sharing of this valuable resource and help disseminate the messages in an area where the number of people with mobile phones is rapidly increasing.

A number of challenges were faced during the development of the DVD. The videos were recorded in Kwaio language and initially had Solomon Islands Pijin sub-titles. The sub-titles were subsequently changed to English, as Solomon Pijin does not have a uniform standard for spelling, can be long when written and could not be easily displayed within the dimensions of the screen. English is the language in which students are formally educated and learn to read and write. Solomon Islands Pijin is primarily a spoken language. Other challenges in the development and screening of the DVD included: the small size of the screen of the portable players; the level of the sound in an open village setting; and charging the battery from solar panels on rainy days when there was little sun. Locally appropriate solutions were applied to each challenge and the DVD was successfully screened.

The project and evaluation have some important weaknesses to acknowledge. The participants in the evaluation are not representative of all people in East Kwaio, so caution must be taken in interpreting the results. The health service leaders and community leaders involved with the project are involved with a learn-by-doing approach to research, as a consequence the methods used may have not been as rigorous as a more experienced researcher would have used. The setting itself in some ways is unique but in many ways is similar to other remote settings across the Pacific.

As a result of the project and evaluation it is recommended that culturally appropriate community-based TB treatment models and supporting health promotion resources be developed and trialed in this setting. In addition the DVD should be made available in the most commonly used language of Solomon Islands – Solomon Islands Pijin. This would enable other communities to see and hear what has been completed in East Kwaio and consider whether something similar could be developed in that local setting. There is a slow increase in the number of DVD players in the area, but the use of mobile technology that can display videos is increasing rapidly.

## Conclusion

The grass roots approach to improving TB control in East Kwaio has strengthened community engagement and resulted in the development of a valuable resource in the local Kwaio language. The local resource was developed within local cultures and based on the oral tradition of Kwaio people. Using modern, but accessible, DVD technology in this developing setting has generated a lot of interest.

The evaluation of the project showed that people generally had a moderate to high level of knowledge about some aspects of TB, and the DVD helped to improve this further. The previously reported delays in seeking treatment due to socio-cultural barriers may be more important than general awareness of TB. Though significant levels of some misconceptions were found, including the belief that TB is caused by sorcery, and that medications can be ceased when a person felt better. The belief that TB is caused by sorcery, and the practice of stopping medications, will be more closely considered during the next stages of the project. The next stages will include an additional DVD and trialing new community-based models of care for TB. The strength of the DVD resource is that it was developed with respect for Kwaio culture and Kwaio language. This DVD is now being used to communicate vital information about what TB is, TB symptoms and treatment in the local Kwaio language. The process of developing community-based audio-visual resources about TB, in local language, and then using the DVDs facilitates stronger community engagement. The stronger community engagement with the health services then provides a pathway for further health improvements.
